# Subjective assessment of brexpiprazole in patients with schizophrenia: a prospective observational study

**DOI:** 10.20407/fmj.2022-031

**Published:** 2023-05-09

**Authors:** Rina Yokoi, Masakazu Hatano, Hiroyuki Kamei, Aoi Morita, Manako Hanya, Nakao Iwata, Shigeki Yamada

**Affiliations:** 1 Department of Pharmacotherapeutics and Informatics, Fujita Health University, School of Medicine, Toyoake, Aichi, Japan; 2 Office of Clinical Pharmacy Practice and Health Care Management, Faculty of Pharmacy, Meijo University, Nagoya, Aichi, Japan; 3 Department of Psychiatry, Fujita Health University, School of Medicine, Toyoake, Aichi, Japan

**Keywords:** Subjective well-being, Drug attitude, Brexpiprazole, Antipsychotic agents, Schizophrenia

## Abstract

**Objectives:**

To investigate the subjective assessments of an antipsychotic treatment with brexpiprazole.

**Methods:**

This was a 14-week prospective observational study. Nineteen patients participated in the study between February 2019 and January 2020.

**Results:**

Patients had a mean age of 40.6±14.2 years and a Clinical Global Impressions-Severity of Illness scale (CGI-S) score of 4.6±1.2 at the initiation of brexpiprazole treatment. The Subjective Well-being under Neuroleptic drug treatment Short form, Japanese version (SWNS-J) total score significantly improved from 68.1±22.3 in week 2 to 79.5±21.0 in week 14 (p=0.0084). The SWNS-J subscales of self-control and social integration status also significantly improved from 14.0±4.7 and 13.9±6.0 in week 2 to 17.0±4.7 and 16.0±5.1 in week 14, respectively (p=0.0053 and 0.012, respectively). No significant improvements were observed in any other SWNS-J subscales or the Drug Attitude Inventory-10 (DAI-10) in the 14-week observation period. Moreover, the SWNS-J total score did not correlate with the DAI-10 (r=0.31, p=0.19), or CGI-S (r=–0.18, p=0.47) scores.

**Conclusions:**

The present results suggest that brexpiprazole might improve subjective well-being, although this may not necessarily reflect psychopathological improvements. To enhance medication adherence, it is important to perform subjective assessments on patients over time.

## Introduction

The treatment of schizophrenia mainly involves pharmacotherapy with antipsychotic agents. Second-generation antipsychotics have been developed that reduce extrapyramidal symptoms (EPS) more than conventional drugs; a meta-analysis showed lower treatment discontinuation and less EPS.^1^ Brexpiprazole was approved by the US Food and Drug Administration in July 2015 as a second-generation antipsychotic with a novel pharmacological profile. Its mechanism of action is not only as a partial agonist of dopamine D_2_ receptors, but also as a partial agonist of serotonin 5-HT_1A_ receptors and an antagonist of serotonin 5-HT_2A_ and adrenergic α_1B/2C_ receptors.^2^ Brexpiprazole is characterized by less intrinsic activity at dopamine D_2_ receptors than aripiprazole. In a recent network meta-analysis, the clinical characteristics of brexpiprazole included a lower risk of akathisia than risperidone and first-generation antipsychotics (e.g., haloperidol), as well as a lower risk of sedation than quetiapine, clozapine, and chlorpromazine.^3^

These superior safety profiles are one of the most important factors for the continuation of antipsychotic treatment, and may contribute to better medication adherence. To achieve good medication adherence, the subjective assessments of patients need to be considered in addition to objective assessments, such as of efficacy and safety. Naber et al. developed the Subjective Well-being under Neuroleptic treatment (SWN) model, which consists of five dimensions: emotional regulation, self-control, mental functioning, social integration, and physical functioning.^4^ Furthermore the Drug Attitude Inventory (DAI) was developed to assess patients’ subjective experiences of treatment, and is used as a predictor of medication adherence.^5^ However, these subjective assessments are rarely considered when new antipsychotic agents are being approved. Brexpiprazole is no exception; the only measures taken into account for its approval were general objective assessments of efficacy (e.g., the Clinical Global Impressions-Severity of Illness scale [CGI-S]) and safety (e.g., cholesterol, glucose, and prolactin).^6,7^ More importantly, although correlations have been reported between subjective and objective assessments, these relationships are not necessarily strong.^8^ Another study identified depression/anxiety as a predictive factor of subjective well-being in the Positive and Negative Syndrome scale.^9^ Therefore, the purpose of the present study was to investigate the subjective assessments of patients receiving antipsychotic treatment with brexpiprazole, and to confirm a relationship between these subjective assessments and conventional objective assessments.

## Methods

### Study design

This was a prospective observational study to assess subjective well-being under brexpiprazole. Subjects were patients administered brexpiprazole for the first time, and the observation period was 14 weeks. Patients eligible for enrollment were aged between 20 and 65 years and diagnosed with schizophrenia according to the definition outlined in the Diagnostic and Statistical Manual of Mental Disorders, Fifth Edition. Patients with contraindications to brexpiprazole were excluded. The dose of brexpiprazole was adjusted by psychiatrists based on the package insert. The present study was conducted at Fujita Health University Hospital between February 2019 and January 2020. Prior to study entry, we adequately explained the study protocol and obtained written informed consent from all patients. This study was approved by the Institutional Review Board of Fujita Health University (HM19-021).

The primary endpoints were mean changes in the SWN Short form, Japanese version (SWNS-J) total score and each subscale score. SWNS-J consists of 20 questions on five subscales (“mental functioning,” “self-control,” “emotional regulation,” “physical functioning,” and “social integration”), each with a six-point scale (1=not at all, 6=very much).^10^ Total scores range between 20 and 120, with higher scores indicating better subjective well-being. Secondary endpoints were mean changes in the DAI-10 total score and subjective global impressions. DAI-10, an abbreviated form of DAI-30, is a scale that assesses patients’ subjective experiences of treatment, and is regarded as a predictor of medication adherence.^5,11^ This scale consists of 10 questions, each with true/false answers; a positive answer is given a score of plus one, while a negative answer is given a score of minus one. Total scores range between –10 and +10, with higher scores indicating a better attitude toward the antipsychotic medication. Subjective global impressions is a self-rating Likert scale with five response categories (1=extremely effective, 2=moderately effective, 3=neither effective nor non-effective, 4=moderately non-effective, 5=extremely non-effective) and is a uniquely designed questionnaire. Other efficacy and safety endpoints were mean changes in the CGI-S and Drug-Induced Extrapyramidal Symptom Scale (DIEPSS). The CGI-S is a seven-point scale (1=normal, not at all ill; 7=among the most extremely ill patients) of the severity of illness at the time of the assessment. DIEPSS evaluates drug-induced extrapyramidal symptoms, which consist of nine items: eight individual items (gait, bradykinesia, sialorrhea, muscle rigidity, tremor, akathisia, dystonia, and dyskinesia) and overall severity, each with a five-point scale.^12^ We also performed a self-reported questionnaire on adverse events at the time of the assessment. At 2, 6, and 14 weeks after the initiation of brexpiprazole, patients were assessed using the SWNS-J, DAI-10, subjective global impressions, CGI-S, and DIEPSS. CGI-S was also assessed at baseline. Moreover, we analyzed the correlations between each rating scale in week14.

### Statistical analysis

Data normality was examined using the Shapiro Wilk test. Changes in the SWNS-J total score, each subscale score, DAI-10, CGI-S, DIEPSS, and subjective global impressions were analyzed using a repeated measures analysis of variance (ANOVA) for a normal distribution or the Friedman test for a non-normal distribution. In the repeated measures ANOVA, Greenhouse Geisser corrections were used when a sphericity assumption was not established. Analyses were conducted using last observation carried forward data. Between-group differences were analyzed using a paired t-test or the Wilcoxon signed-rank test (for normal or non-normal distributions, respectively), with Bonferroni corrections for post-hoc comparisons. The significance level was corrected to p<0.0167 in comparisons of three groups or to p<0.0083 in comparisons of four groups.

The relationships between each rating scale were assessed using Spearman’s rank correlation coefficient. P-values were two-sided and those less than 0.05 were considered significant. The sample size was set as the number of feasible cases during the term because this was a preliminary study. All statistical analyses were conducted using R 3.4.3 (The R Foundation for Statistical Computing).

## Results

### Patient characteristics

Nineteen patients participated in the present study. Patient characteristics are summarized in Table 1. The initiation methods of brexpiprazole were add-on to antipsychotics in 11 patients, switching from antipsychotics in seven patients, and first-episode schizophrenia in one patient. The antipsychotics used by patients prior to the initiation of this study were as follows: aripiprazole (switching: n=3, add-on: n=2), clozapine (add-on: n=5), olanzapine (add-on: n=2), asenapine (add-on: n=2), perospirone (switching: n=2), quetiapine (add-on: n=1), blonanserin (switching: n=1, add on: n=2), risperidone (switching: n=1, add on: n=1), and chlorpromazine (add-on: n=1). At the initiation of brexpiprazole, the mean CGI-S score was 4.6±1.2, indicating moderate illness. All patients continued brexpiprazole for up to 6 weeks; however, six patients had dropped out by 14 weeks for the following reasons: withdrew consent (n=2), adverse event (n=2, akathisia and tremors), exacerbation of disease (n=1), and lost to follow-up (n=1).

### Time course of subjective and objective rating scales

The time course of changes in the SWNS-J (total and subscale scores), DAI-10, disease severity (CGI-S), DIEPSS, and subjective global impressions is shown in Table 2. The SWNS-J total score significantly increased from 68.1±22.3 in week 2 to 79.5±21.0 in week 14 (p=0.0084). The SWNS-J subscales of self-control and social integration status also significantly increased from 14.0±4.7 and 13.9±6.0 in week 2 to 17.0±4.7 and 16.0±5.1 in week 14, respectively (p=0.0053 and 0.012, respectively). In addition, CGI-S scores significantly decreased from 4.6±1.2 at baseline to 3.4±1.1 in week 14 (p=0.0079). No significant improvements were observed in any other SWNS-J subscales (mental functioning, emotional regulation, or physical functioning), DAI-10, DIEPSS, or subjective global impressions between week 2 and weeks 6 and 14.

### Relationships between each rating scale

The relationships between each rating scale in week 14 are shown in Table 3. The SWNS-J total score did not correlate with DAI-10 (r=0.31, p=0.19), CGI-S (r=–0.18, p=0.47), or DIEPSS (r=–0.26, p=0.28) scores. The SWNS-J subscale scores also did not correlate with DAI-10, CGI-S, or DIEPSS scores. However, DAI-10 scores negatively correlated with subjective global impression (r=–0.62, p=0.0043), CGI-S (r=–0.52, p=0.021), and DIEPSS (r=–0.82, p<0.001) scores. Subjective global impressions negatively correlated with the SWNS-J total score (r=–0.60, p=0.0070) and each subscale score except for mental functioning (self-control, r=–0.53, p=0.020; emotional regulation, r=–0.61, p=0.0051; physical functioning, r=–0.49, p=0.032; social integration, r=–0.47, p=0.042).

## Discussion

The present study investigated three subjective assessments (subjective well-being, subjective experiences of treatment, and subjective global impressions) in patients treated with brexpiprazole. Brexpiprazole might improve SWNS-J scores in the 14-week observation period, suggesting efficacy not only for psychopathological improvements, but also for subjective well-being. Previous studies have reported that various antipsychotic treatments can improve patients’ subjective assessments, and the present results support these findings.^13^ Early improvements in subjective well-being are related to symptomatic remission,^14^ and brexpiprazole may similarly contribute to long-term remission. In contrast, DAI-10 scores did not show significant improvements within the observation period in the current study. Although few studies have evaluated DAI over time during antipsychotic treatments, recent studies have reported improvements with paliperidone.^15^ Because the present study also comprehensively evaluated untreated patients, we were unable to measure each subjective score at the initiation of brexpiprazole. Therefore, changes in each score within 2 weeks of the initiation of treatment were not reflected; as a result, the effect size may have been underestimated. This is also the case for the SWNS-J evaluation.

Psychiatric symptoms might improve over the course of treatment, but CGI-S scores did not correlate with SWNS-J total scores or each subscale score. Scores in the SWN haves been reported to only moderately correlate with psychopathology, with positive scores being more weakly associated than negative or global scores.^16^ However, the present investigation was a preliminary study, with a relatively small sample size; thus, the results obtained may have been false negatives because of the low effect size. Furthermore, the severity of illness was moderate at baseline and 18 out of the 19 patients receiving outpatient treatment were relatively stable, which may have contributed to the small psychopathological changes that were observed with the antipsychotic administration. Subjective well-being does not necessarily correlate with improved psychopathology, and any relationship is likely to be small. Conversely, DAI-10 scores moderately correlated with CGI-S scores in the present study. A significant correlation between DAI scores and severity of psychopathology was observed in a cross-sectional study of more than 200 patients, and our results corroborate previous findings.^17^

Notably, subjective global impression scores did not significantly improve within the observation period, and did not correlate with psychopathology. Subjective global impressions were assessed in the present study using a Likert scale, similar to CGI-S, and were rated by patients themselves and not by clinicians. In a previous study in which clinicians and patients both rated CGI-S, there were moderate correlations between scores; however, clinician-rated CGI-S scores were strongly associated with positive symptoms whereas patient-rated CGI-S scores were associated with depressive symptoms.^18^ Although the present study did not assess psychiatric symptoms in detail, depressive symptoms did not appear to sufficiently improve to positive symptoms. Thus, although subjective global impression scores and CGI-S scores are similar Likert scales, their implications may not necessarily be consistent between patients and clinicians; and caution is needed when interpreting the results. In addition, subjective global impression scores correlated significantly with SWNS-J and DAI-10 scores in the present study, and patient-assessed measurements using the Likert scale may therefore be useful for the subjective assessment of treatments.

Although we focused on brexpiprazole, the effects of different pharmacological profiles of antipsychotics on subjective well-being have not yet been clarified. No significant differences were observed in comparisons between olanzapine and risperidone.^19^ Regarding comparisons among long-acting injections, aripiprazole showed a slight numerical improvement over paliperidone.^20^ In addition, contradictory findings have been reported for improvements in subjective well-being; with second-generation antipsychotics have been reported as superior to or no different from first-generation antipsychotics.^13^ In one study, high-dose first-generation antipsychotics were related to negative subjective well-being, whereas second-generation antipsychotics did not show dose-dependent impairments; the researchers suggested the influence of concomitantly administered anti-Parkinson agents.^21^ Subjective well-being has been shown to correlate with extrapyramidal symptoms^22^ and sexual dysfunction.^23^ In contrast the present subjective well-being results did not correlate with DIEPSS scores. This discrepancy may have occurred because the majority of participants were not using first-generation antipsychotics at baseline.

The present study had several limitations. The sample size was small because it was a preliminary investigation limited to brexpiprazole; thus, its statistical power was limited. A previous study that evaluated SWN over time calculated that 30 patients were needed to provide 80% statistical power.^24^ We must therefore, consider the possibility that some results were false negatives. We were also unable to assess differences in patient baselines because of insufficient statistical power. In particular, 5 of the 19 patients had treatment-resistant schizophrenia using clozapine and may have been poor responders to brexpiprazole. In addition, differences in the initiation methods (add-on or switching) of brexpiprazole and concomitant medications (e.g., benzodiazepines and anti-Parkinson drugs) need to be considered in future studies. We were also unable to examine the effects of the concomitant use of benzodiazepines or anti-Parkinson drugs on the results.

In conclusion, the present results suggest that antipsychotic treatment with brexpiprazole might improve not only psychopathology, but also subjective well-being. However, improvements in psychopathology in particular, positive symptoms were not necessarily reflected in improved subjective well-being. The SWN is independent of objective assessments conducted by clinicians, and evaluations over time may lead to better patient adherence to medication.

## Figures and Tables

**Table1:** Baseline patient characteristics

	Patients (n=19)
Age (yr), mean±SD	40.6±14.2
Male sex, n (%)	12 (63.2)
Disease duration (yr), mean±SD	11.0±8.4
Married, n (%)	5 (26.3)
Employed, n (%)	7 (36.8)
Outpatients, n (%)	18 (94.7)
Chlorpromazine equivalent (mg), mean±SD	575.5±324.6
Diazepam equivalent (mg), mean±SD	9.0±14.9
Biperiden equivalent (mg), mean±SD	0.7±2.3
CGI-S, mean±SD	4.6±1.2

Abbreviations: CGI-S, Clinical Global Impressions - severity of illness

**Table2:** Time course of subjective and objective rating scales

	Baseline mean±SD	Week 2 mean±SD	Week 6 mean±SD	Week 14 mean±SD	Repeated measures ANOVA			Friedman test			Group comparisons, *p* value		
					F	*p* value		X^2^	*p* value		Baseline vs. Week 2	Baseline vs. Week 6	Baseline vs. Week 14
CGI-S	4.6±1.2	3.8±1.0	3.5±1.1	3.4±1.1	—^b^	—^b^		15.1	0.0018		0.014	0.0098	**0.0079**
SWNS-J													
total	—^a^	68.1±22.3	76.4±20.4	79.5±21.0	6.21	0.0048		—^b^	—^b^		—^b^	0.037^c^	**0.0084^c^**
mental functioning	—^a^	13.4±5.2	15.3±3.8	15.6±5.2	3.92	0.029		—^b^	—^b^		—^b^	0.053^c^	0.033^c^
self-control	—^a^	14.0±4.7	16.3±4.4	17.0±4.7	7.43	0.0052		—^b^	—^b^		—^b^	0.019^c^	**0.0053^c^**
emotional regulation	—^a^	13.7±5.0	14.7±4.3	15.7±4.2	—^b^	—^b^		2.80	0.25		—^b^	0.27^c^	0.041^c^
physical functioning	—^a^	13.2±4.6	14.0±5.3	15.2±5.1	2.34	0.13		—^b^	—^b^		—^b^	0.44^c^	0.10^c^
social integration	—^a^	13.9±6.0	16.1±5.5	16.0±5.1	5.72	0.0069		—^b^	—^b^		—^b^	0.021^c^	**0.012^c^**
DAI-10	—^a^	5.2±3.8	5.6±4.7	6.2±4.2	—^b^	—^b^		2.00	0.37		—^b^	0.69^c^	0.20^c^
DIEPSS	—^a^	3.4±3.4	2.0±2.7	1.7±2.8	—^b^	—^b^		3.66	0.16		—^b^	0.044^c^	0.032^c^
Subjective global impressions	—^a^	2.3±0.9	2.0±0.8	2.0±0.8	—^b^	—^b^		4.67	0.097		—^b^	0.15^c^	0.071^c^

Abbreviations: CGI-S, Clinical Global Impressions - severity of illness; SWNS-J, Subjective Well-being under Neuroleptic drug treatment Short form, Japanese version; DAI-10, Drug Attitude Inventory-10; DIEPSS, Drug Induced Extrapyramidal symptom scales ^a^ Not measured at baseline ^b^ Not analyzed ^c^ Comparison with week 2

**Table3:** Correlation of each rating scale in week 14

	DAI-10	Subjective global impressions	CGI-S	DIEPSS
SWNS-J, *r* (*p*-value)				
total	0.31 (0.19)	**–0.60 (0.0070)**	–0.18 (0.47)	–0.26 (0.28)
mental functioning	0.40 (0.093)	–0.40 (0.092)	–0.16 (0.52)	–0.37 (0.12)
self-control	0.42 (0.073)	**–0.53 (0.020)**	–0.22 (0.36)	–0.29 (0.24)
emotional regulation	0.30 (0.21)	**–0.61 (0.0051)**	–0.19 (0.44)	–0.32 (0.18)
physical functioning	0.32 (0.19)	**–0.49 (0.032)**	–0.20 (0.42)	–0.30 (0.22)
social integration	0.12 (0.62)	**–0.47 (0.042)**	0.024 (0.92)	0.0091 (0.97)
DAI-10, *r* (*p*-value)	—	**–0.62 (0.0043)**	**–0.52 (0.021)**	**–0.82 (<0.001)**
Subjective global impressions, *r* (*p*-value)	—	—	0.25 (0.30)	0.35 (0.14)

Abbreviations: SWNS-J, Subjective Well-being under Neuroleptic drug treatment Short form, Japanese version; DAI-10, Drug Attitude Inventory-10; CGI-S, Clinical Global Impressions - severity of illness; DIEPSS, Drug Induced Extrapyramidal symptom scales
